# The Possibility/Impossibility of Ethical Community during the COVID-19 Pandemic: a Philosophical Reflection

**DOI:** 10.1007/s41649-021-00168-0

**Published:** 2021-03-13

**Authors:** Shining Star Lyngdoh

**Affiliations:** Manipal Centre for Humanities, Manipal Academy of Higher Education, Manipal, Karnataka India

**Keywords:** Aporetic community, Ethics towards the other, Nancy, Levinas

## Abstract

The outbreak of COVID-19 has raised a global concern and calls for an urgent response. During this perpetual time of epidemic crisis, philosophy has to stand on trial and provide a responsible justification for how it is still relevant and can be of used during this global crisis. In such a time of crisis like that of COVID-19, this paper offers a philosophical reflection from within the possibility/impossibility of community thinking in India, and the demand for an ethical responsivity and response-ability to act ethically towards the Other (*autrui*) to show that philosophy always already emerges from within the context of crisis. As an alternative outlook to the thinking of totalitarian singularity and individualism, community—in its possible and impossible making—can offer more meaningful engagement with the other human being by being responsible and extending care towards the Other. The thinking of a shared community life is the facticity of one’s own being-together-in-common without the dismissal of individual differences as can be seen in the works of Jean-Luc Nancy, and there is an ethical demand that comes from the face-to-face ethical relationship with the Other as argued by Emmanuel Levinas.

## Introduction

The early decades of the twenty-first century seem to have their own global crisis, which could be different from that of the twentieth century. During this time of crisis, the question of the role and the use of philosophy (as a distinct academic discipline) always pops up, and such a question has been raised not only by those who call themselves silent spectators from a distance, but more importantly, by those whom we consider the practitioners of philosophy. The question is always about the speculation on the public and practical relevancy of philosophy. And, during the global COVID-19 pandemic, philosophy again has to confront similar question as it has been doing in the past.^1^ In the context of the present scenario, the question can be raised as where is the place of theoretical-philosophical thinking during the perpetual time of epidemic crisis and the practical utility of such a discipline. This question, guided by instrumental rationality, aims to arrive at the possibility of proving the utility of philosophy. If philosophy is to have a significant practical contribution, it has to understand its role in unravelling the existential question of who we are today, the understanding of one’s finitude and the crisis of a community rather than continuing the ongoing search for an abstract universal truth and foundation that function as a transcendental truth.

The obstruction of the coronavirus pandemic has compelled philosophy to reflect and rethink the existential crisis that is prevalent in the community life of collective individuals—be it of healthcare professionals, tertiary sectors, and other service providers—and to address the problem of ethical dilemma faced within a given community of sharing.^2^ Within communitarian thinking, humans were seduced to reflect upon their existence and life-world in a manner that they could not have thought before. The global crisis (in all senses of the term) has demanded a rethinking and reimagining of various domains of human existence—be it that of the socio-politico-theological, the advancement of healthcare facilities, the availability of human resources, or the strength of national economy and security. This exigency is for a *retrait*—both as withdrawing and as retracing—that draws philosophers’ attention to reflect upon the existential question of community and of a shared communitarian life. The question of human relationship within a community is one of those areas, which has been an oversight in contemporary times with the rise of individualistic thinking and the embracing of a totalitarian individual autonomy based on a Kantian self and a Cartesian model of an independent *cogito*.

There is a popular imagination of an individual self as an egocentric self, completed and sufficient within the same self and independent from other human beings’ assistance. On the other hand, there is an argument that the self is incomplete and has no essence without the other. There are infinite traces of alterity other than that of the self on which the same self has to depend upon and the egocentric self is incomplete without the traces of other human beings. The self’s subjectivity is situated not in its totalizing and comprehensive understanding but in its radical relation to the alterity of not the same (or the other human being) and the irreducibility of the other individual to the same (or the “I”). This is to emphasize that “one cannot make a world with simple atoms. There has to be a *clinamen*. There has to be an inclination or an inclining from one towards the other, of one by the other, or from one to the other” (Nancy 1991, 3). To state it more radically, it is to argue that there is no self without the infinite traces of the other. Such debate can be viewed as that of individualism and communitarianism, and it is one of the significant refections to relook during the COVID-19 pandemic.

By drawing certain philosophical insights from Jean-Luc Nancy’s thinking on community as the “*clinamen* of the ‘individual’” as in his work *The Inoperative Community* (Nancy 1991, 4) and Emmanuel Levinas’ ethics towards the Other,^3^ as explicated in his *Totality and Infinity: An Essay on Exteriority* (Levinas 1979), this paper aims to provide a philosophical reflection on the idea of the possibility/impossibility of the making and unmaking of community and communitarian life during/(post-) COVID-19 pandemic from within the ethical stance of responsivity, response-ability (read as responsibility) and care towards the Other in a diverse country like India. Even though there have been voices on the question of economic and health-related issues, and the relation of medical ethics with the COVID-19 pandemic in India, the thinking of ethical community from within a philosophical reflection has not received due attention in academic writings. (However, this paper is not confined to the prevailing scenario in India but to the world at large, keeping in mind the common precarious life faced by various communities of individuals at a global level). It is also important to note that the philosophical ideas of this paper can be applied not only to a non-totalizing community as a whole but to multiple groups of communities like that of medical doctors, nurses, paramedics, and other sections of community members who are at the frontline of combating the spread of COVID-19. The philosophical thinking and writing of Nancy and Levinas focus on related themes of the question concerning the relation of an individual self and the Other within the imagination of communitarian life. The question that can be raised here is: where is the place of communitarian thinking and ethical responsibility during the outbreak of the coronavirus pandemic in India and across the globe?

## Community and COVID-19

The thinking of community and communitarian life during/(post-) pandemic crisis of coronavirus in India stands for the need and desire to relook the missing place of community living beyond the social and economic differences, division, and segregation inherited from time immemorial. Individualistic thinking guided by social division tends to be a preferable lifestyle and tends to forget the infinite traces of the other human beings in a community. India’s contemporary society manifests its own face of separation and isolation and embraces a form of life that promotes individuality and alienation. Further, it is not an exaggeration to say that human society has been populated by selfish individuals who only think about themselves first, and communitarian living is only a matter of convenient practices. The outcome of such ways of living can lead to a disintegrated society of violence. The absolute self or the “I” projects himself or herself in his/her own totality and ignores the infinite traces of the other, as argued by Levinas. This suggests that the called for “being-with” as a community to experience the shared communitarian life for human beings is defined in it being relational and not in its absoluteness and totality. The call can be of ethical responsibility towards the other, othered by social inequality, caste, creed, or gender. An ethical response-ability is the existential need of a community during the pandemic crisis.

The idea of community as the understanding of “being-in-common” as espoused by Nancy takes us to the realization of the finitudeness of human existence and the interdependence on other members within the same community and those outside the given community. The conceptualization of human finiteness and its realization bring together the idea of human beings as always already “being-together-with” in common and practice the sacrificial act of sharing within a given community. The communitarian life of being together in common and sharing (and being hospitable towards the Other) brings forth its essence of communitarian life for a community presupposed to possess a shared social knowledge of being human. To be *human* within a community is to share the essence of communitarian living together in a non-essentialistic formulation, conditioned by social equality and respect for being a human. The integration of the essence of being human is important for living a communitarian life. This is the exigency from within the essence of a shared community life.

In participating in the act of sharing as being together in common, the individual is neither the receiver nor the giver. The act of sharing within a community violently destabilizes any bipolarities while recognizing a recipient and a giver’s individuality. In such an action, sharing does not give a linguistic description of who we are or what we do for sharing is itself the very essence of our being human. To put it in the words of Nancy: “[i]f being is sharing, *our* sharing, then ‘to be’ (to exist) is to share” (Nancy 1993, 72). The act of sharing fails to have any form of a linguistic description as it goes beyond semantic use of language to that of ontological existence. It is such an act of sharing that entails the constitutive and deconstitutive play of the demand for community life. In other words, sharing can make and unmake a community. Sharing is the manner in which the community presents herself to the singularity of my individual existence. It is in sharing that the individual self could recognize what a community is.

In the thinking of community, it is to be pointed out that the ideal claim of a shared community life is not to deny the reality of individual differences and diversity. The conceptualization of a community without the autonomy (in a non-Kantian understanding of the term) of an individual self cannot be conceived. Further, an individual self cannot be reduced to the total absoluteness of a community. As argued by Nancy, the meaningful interpretation of a shared community life is not to be found in the imagination of “common being” or the same being like “me,” but in the facticity of our “being-in-common” in the midst of infinite differences (Nancy 1991, 29). Such diversities and differences need to be recognizable and embraced in a shared community life for building mutual love and respect among its members.

To argue further, there is a demand for a kind of resistance to the absolute singularity of an individual without the need of reducing the unique individuality of that individual. Such is the manner that can be considered as the hallmark of a communitarian life for the fusion of the infinite traces of differences is not to be taken as a threat to the thinking of the community itself. This is to prevent uniformity, and a homogenous mode of thinking within the non-totalizing community for the goal of a shared community life is not to create a unilateral culture within the community by reducing the essence of an individual subject. Such a community would be “... a nontotalizing community, a community which recognizes that we do not need to have everything in common, that our differences communicate, but they communicate in such a way as to recognize the abyss or gulf of singularity, the idiosyncrasy of the singular, the irreducible, untranslatable idiomatic quality of the singular” (Caputo 1996, 26). Differences and fluidity of understanding have to be considered while thinking about the possible/impossible making and unmaking of a non-totalizing community. To put it in other words, it is from the facticity of a shared community life that emerges varieties of diversities, differences, and non-homogeneity of singularities. Community, therefore, shows the impossibility of its project of the common and homogenous community as it projects the rupture of its own *retrait*. Such thinking of community is hostility to its ideal.

However, it is essential to assert the limit of the thinking of the possibility/impossibility of the making of the community, for it carries within itself the mark of its own limit that emerges from within its own horizon. This is to argue that there are breakdown and obstruction within the thinking of community itself. The community has no community of homogeneity and no contractarian consensus that lies within the community. In this manner, the claim is not that of a general agreement on a nostalgic longing for the lost idealistic communitarian and *praxis* nor an attempt to reconstitute or reclaim that community. In addition, communitarian thinking during a pandemic crisis is not a reflection for what was lost. This is to argue that the thinking of community is not that of romanticization of the past nor that of a utopianistic project of reclaiming the past. The thinking of community is of the frictional play operated within the community itself in the present/absent of the here and now. Neither is the image of the past nor that of anticipation in the near future is the thinking of the community. The thinking of community is not a nostalgic image for what has been considered as a long lost golden value of the past that has to be restored, and it is not the utopianistic projection of a community to come, but of a shared community of the here and now, which can emerge from within the thinking of the community. However, the here and now of communitarian thinking is always already an ongoing struggle of a shared community life. In this manner, one can argue that the sense of a community is always in the process of ongoing thinking. The ideal of shared community life is never fully achievable because it has no fixity of meaning as it cannot have a universal meaning. The thinking of community life is the opening to its fluidity for its resistance to all kinds of homogeneity, totalitarian imagination of a community, and the fusion of a common identity. It resists meaningful description as it entails the presence/absence of a foundation. In this manner, it is always an ongoing project of the here and now in the in/outside of the context of foundational ethics.

## Ethical Response-ability

In the light of such thinking on a shared community life without the desire for totalizing the differential community, the resistance to the fetishism of singularity and the recognition of the limits of community, the desirability for a shared community life in the midst of popularizing physical (“social”) distancing during the COVID-19 pandemic crisis (in India) emerges from within the necessity of the essence (in a non-essentialistic claim) of human existence, which is the facticity of “being-together-with” within a shared community. In the act of a meaningful sharing, there is the making and unmaking of communitarian life, for the important play is that of sharing and not that of constructing the autonomy of absolute singularity. A shared communitarian life is to recognize and accept differences and diversities without the need to reduce the other human being into the totalitarian imagination of the individual self. In other words, the essence of communitarian life lies not in the fusion of a single identity but in exposing, recognizing, and encountering that infinite trace of differences and exteriority of the Other as argued by Levinas in his *Totality and Infinity* (Levinas 1979, 194–197). Shared community life is a community that critically welcomes all sets of differences and diversities of being-in-common and not that aspires the commonness or any mode of totalizing singularity. A totalitarian fusion of oneness and commonness can be a threat to the possibility of diversity and difference. A totalitarian and singularity community is not a communion of being together, displaying a shared communitarian life. This reflects the common character of human existence that gave rise to our being in common with each other and “being-together-with” along with the fusion of distinct differences. To put it in other words, this is the making and unmaking of a fortified community along with the strong integration of quasi-identitarian differences. In this way of thinking community, there is a co-habitation of separation and difference within a collective relation of the interconnectedness of autonomous individuals.

The way of invoking the demand of communitarian thinking and *praxis* during the time of the pandemic crisis in India emerges from within a tradition that witnesses the violence of economic injustice and social inequality. And, during the pandemic crisis of coronavirus in India and the imposition of national lockdown that led to the financial instability of those belonging to a ‘community without community’, they have to undergo severe difficulties for such a community is always identified as a marginalized and underprivileged community.^4^ They belong to a community that has no community of sharing and caring, a community of negation and deprivation. This community is always unorganized and considered as a deprecated community, which has to face undeniable violence. It was such a community that has to suffer while applying the utilitarianistic formula, making a sacrifice for the sake of the majoritarian community. In such a scenario, there is the operation of the inclusion and exclusion within a community and particularly in such a community like that of the absence of a shared communitarian life.^5^

Keeping the above social reality in mind, the exigency of a shared community life during/(post-) COVID-19 is always an ethical demand for response-ability and responsivity towards the call of a community in a manner of being open-ended towards the ethical demand of the Other. For a community thinking to be meaningful, it has to go beyond itself to the “community without a community”. This is the demand for being ethical and being sensitive to such a community and recognizing the ethical responsibility of inter-subjectivity. In this manner, one can argue that the ground of ethical action is no longer centered on the self, but in this social interaction with the other human beings within a community of shared life-world as propagated by Levinas. This is also to argue that ethical responsibility cannot be isolated from the meaningful thinking of a community because it holds us responsible and navigates our ethical action in a community.

The claim that ethical responsibility towards a “community without community” is to argue that being hospitable and care or ethical relation towards the Other can be considered the groundless ground or basis of “being-together-with” and participating in a shared community life. It is the Other who calls us to act ethically towards them, to be otherwise that the same,^6^ and to go beyond the self by barring all possible conditions and antinomy. In responding to the call of the Other, the self can be an ethical other, and this is not to argue that the Other can take away the freedom and autonomy of the self. It is in the primordial relation with the Other, the asymmetrical relation between the self and the Other that makes the subject (self) to see the need to respond to the cry of injustice against the Other. Such an ethical relation of the self with the Other and the implicit call to responsible action is not that the subject could discover or invent. Rather, it is always already there in the inherent nature of this ethical relation. It is this ethical responsibility, and responsivity to those whom we have no direct social or kin relationship with that upholds a community of being with each other in the midst of diversities and differences. It is here that there is a demand for destabilizing the primacy and totality of the self and move towards the understanding of the Other ethically. In the encountering of the Other, one can experience the closed relationship of being together within a community of a shared life-world. In other words, the experiential facticity of a shared community life lies in the ethical (face-to-face) relation with the Other, as argued by Levinas.

Further, one can argue that Levinasian methodical inquiry of ethics (considered as first philosophy) that transcends beyond human nature and essence and beyond socio-ethnic bipolarities provides a suitable lens to understand ethical relations in a community of being-together-with. The Levinasian model of ethics provides us with a perspective of understanding the human experience and the everydayness existence of those belonging to a community without community during social exclusion. In this thinking, there is no exteriorization of the Other. The Other is the absolute other, which cannot be contained and reduced to any form of description, and in the encounter with the Other, there is no violent exteriorization of the Other outside her/his shared community life. It is in the primordial ethical relation with the Other who always invites and calls the subject self to act ethically responsibly that one can argue for the possibility/impossibility of a shared communitarian life during/(post-) the outbreak of COVID-19. In the face-to-face relation (which is an ethical relation), the Other is not taken as the other human being but as belonging to a community without reducing the Other to the totality of the same.

As discussed above, the exigency of experiencing a shared community life is an ethical demand for response-ability and care for the Other. This is to argue that the call for retracing the idea of a shared community life of being together in common is primarily an ethical demand in the manner of supplication and humility and not out of violence and conflict. The experience of a shared community life emerges from within the response to the demand of ethical response-ability (responsibility) of its own members in their participation within their own community’s life world. Without the ethical responsibility of the self towards the Other and being hospitable towards the Other, the thinking of community is redundant. The making-unmaking of a community is strongly connected to the ethical thinking of being responsible towards the Other. In other words, for a shared community life to be made possible, the sense of being together with the Other is an ethical claim of what we are as finite beings. With such an understanding, there is a strong exigency for such ethics of response-ability (responsibility) and responsivity towards the Other in a shared community life during the perpetual time of crisis.

## Concluding Remarks

In conclusion, it is important to revisit the question of relevancy and contribution of philosophical thinking during/(post-) COVID-19 as raised in the introduction of this paper. Throughout the paper, the argument is to show that a contribution of philosophical reflection on community and ethical responsibility towards the Other can be a plausible or alternative way of understanding human relation during the time of crisis. This can be one way of reflecting on the importance of community thinking and ethical responsibility among other contributions of philosophical thinking. Such contribution is to be formulated in a non-condescending, non-depredating manner without being nostalgic, longing for the reconstituting the community of the past, while offering an alternative view of imagining ethical community outside the totalitarian thinking.

The upshot of this paper is the possibility/impossibility of the making and unmaking of a shared community by understanding the ethical demand for being hospitable and responsible towards the Other during the outbreak of COVID-19 in India. The argument is that of philosophical speculation and reflection on the social scene in India’s community living. The philosophical reflection of Nancy’s communitarian living and the Levinasian thought on ethical responsibility towards the Other emerges as a way of understanding ethical community during epidemic crisis.

## Footnotes

1 The western canonical philosophical tradition reveals to us that philosophy always has to respond to socio-political, pandemic, economic crises at various historical epochs and new philosophical ideas have always emerged from within the context of various external crises or from within the crisis of philosophy itself.

2 This could be one challenge among many other issues that demand our attention, and this paper is focusing only on the possibility/impossibility thinking of ethical community life during the crisis of the COVID-19 pandemic.

3 Throughout this paper, the term Other (*autrui*), with a capital “O” has been used to refer to other human being, and the term other (*autre*), with a small “o” is use to refer to anything other than living human being. This is in line with the Levinasian usage of the term in the English translation of Levinas’ corpus.

4 https://www.thehindu.com/news/national/coronavirus-lockdown-26-lakh-migrant-workers-in-halfway-houses-says-official-data/article31751222.ece. (Accessed 31 August 2020). According to media reports, large groups of migrant workers had to walk on foot to reach their village.

5 To convey the radicality of the thinking of community, is it not the conceptualization of community that always has to be imagined as having both an inside and an outside of a community? Is not community itself that suggests the inclusion and exclusion of its own members, and that it can go beyond itself? There is the operation of the possibility and impossibility of the making and unmaking of the community. The aporetic community is at play in its non-structural operation.

6 The usage of the term “same”, understood as the self or the “I”, is borrowed from Levinas’ works.
